# A229 DRUG PERSISTENCE WITH THERAPY OF ADALIMUMAB BIOSIMILAR, IDACIO, IN INFLAMMATORY BOWEL DISEASES

**DOI:** 10.1093/jcag/gwad061.229

**Published:** 2024-02-14

**Authors:** S Cheung, F Kudaeva, P Heidari, D Truong

**Affiliations:** Medical Affairs, Fresenius Kabi Canada Ltd, Toronto, ON, Canada; Medical Affairs, Fresenius Kabi Canada Ltd, Toronto, ON, Canada; Medical Affairs, Fresenius Kabi Canada Ltd, Toronto, ON, Canada; Medical Affairs, Fresenius Kabi Canada Ltd, Toronto, ON, Canada

## Abstract

**Background:**

For post marketed medicine, drug persistence could be used as an indication for effectiveness and safety in routine clinical care over time. Non-adherence and non-persistence to treatment are associated with disease flares and may lead to disease progression among patients with inflammatory bowel diseases (IBD) in real-life.

**Aims:**

The aim of this analysis was to explore drug persistence with an adalimumab biosimilar, Idacio, in IBD patients using the data collected by Fresenius Kabi patient support program (KabiCare) in Canada.

**Methods:**

Patients with IBD enrolled in KabiCare and received at least one dose of adalimumab biosimilar, Idacio, were followed up from February 2021 to September 2023 in Canada. Medication initiation date and stop date, disease types (Crohn’s disease [CD], and ulcerative colitis [UC]) and patient treatment (categorized by naïve to biologic,or switched from other biologics) were recorded in the database. The stop date was the date when KabiCare was informed that medication was discontinued. The patients continuing medication were censored at the maximal follow-up date of analysis, September 12, 2022. Log-rank tests were used for KM (Kaplan-Meier) plot comparison to examine if there were differences in medication adherence between CD and UC, and between naïve group vs. switch group by disease.

**Results:**

The study sample consisted of 1049 patients: 830 (79.1%) had CD, and 338 (32.2%) were in naïve group. KabiCare followed patients with CD for a median time of 512 days (interquartile range: 285-673 days; maximum: 895 days), and followed patients with UC for a median time of 492 days (interquartile range: 183-672 days; maximum: 925 days). At 800 days after starting the medicine, the drug persistence probability was similar for patients with CD and UC (At day-800: 76.6% vs. 67.6%, p=0.07). Among patients with CD, the drug persistence probability was higher in the switch group compared to naïve group (At day-800: 78.9% vs. 71.7%, p=0.0028). Among patients with UC, the drug persistence probably was similar in the switch group compared to naïve group (At day-800: 78.4% vs. 62.3%, p=0.34).

**Conclusions:**

The drug persistence of adalimumab biosimilar, Idacio, was over 76% in patients with CD and over 67% in patients with UC 800 day after therapy initiation. Compared to patients in the naïve group, patients in the switch group were more likely to stay on adalimumab therapy.

**Funding Agencies:**

None

